# Postoperative Natural Killer Cell Dysfunction: The Prime Suspect in the Case of Metastasis Following Curative Cancer Surgery

**DOI:** 10.3390/ijms222111378

**Published:** 2021-10-21

**Authors:** Marisa Market, Gayashan Tennakoon, Rebecca C. Auer

**Affiliations:** 1 Faculty of Medicine, University of Ottawa, Ottawa, ON K1G 8M5, Canada; mmark030@uottawa.ca (M.M.); gtennakoon@ohri.ca (G.T.); 2 The Ottawa Hospital Research Institute, Ottawa, ON K1G 4E3, Canada; 3 Department of General Surgery, The Ottawa Hospital, Ottawa, ON K1Y 4E9, Canada

**Keywords:** surgery, natural killer cells, immune suppression, cellular immunity, cancer

## Abstract

Surgical resection is the foundation for the curative treatment of solid tumors. However, metastatic recurrence due to the difficulty in eradicating micrometastases remain a feared outcome. Paradoxically, despite the beneficial effects of surgical removal of the primary tumor, the physiological stress resulting from surgical trauma serves to promote cancer recurrence and metastasis. The postoperative environment suppresses critical anti-tumor immune effector cells, including Natural Killer (NK) cells. The literature suggests that NK cells are critical mediators in the formation of metastases immediately following surgery. The following review will highlight the mechanisms that promote the formation of micrometastases by directly or indirectly inducing NK cell suppression following surgery. These include tissue hypoxia, neuroendocrine activation, hypercoagulation, the pro-inflammatory phase, and the anti-inflammatory phase. Perioperative therapeutic strategies designed to prevent or reverse NK cell dysfunction will also be examined for their potential to improve cancer outcomes by preventing surgery-induced metastases.

## 1. Opening Arguments: A Brief History of Cancer Surgery 

Cancer is one of the leading causes of death in North America, with an estimated 1.9 million new cancer cases and 693,000 cancer deaths in 2018 [1]. Despite advances in detection and treatments, the five-year net survival for people diagnosed with cancer in Canada is 60% [2]. Historically, the treatment of cancer has long been a medical challenge, as has been recorded in the earliest chronologies of human existence. Although the word “cancer” was not used, the oldest description of this disease can be found in the Edwin Smith Papyrus, an ancient Egyptian text that dates back to 3000–2500 B.C [3,4,5]. The text describes breast tumors that were surgically removed using primitive tools, such as the “fire drill” [4,5]. These treatments proved futile and this record concludes that “there is no cure” [4,6]. Surgery continued to be provided as a largely unsuccessful treatment for a variety of cancers, until the development of anesthesia in 1846 radically changed the field of surgery, allowing for longer, more complex surgeries [7]. In 1890, William Halsted developed the Halstedian model for cancer progression and proposed that cancer (specifically breast cancer), spreads through the lymphatic system. This resulted in a strong emphasis on aggressive locoregional treatment in an attempt to avoid cancer recurrence [8,9]. There was a rapid development of cancer surgery techniques, ranging from abdominoperineal resection to pneumonectomy, to radical hysterectomy, and radical suprapubic prostatectomy, in the early 20th century [8,10]. Halstedian surgical techniques were later shown to provide little benefit over less invasive procedures and have since been replaced by less radical procedures that have significantly improved patient quality of life [8,11,12]. Today, surgery provides undeniable benefits to cancer patients, as complete surgical resection is curative in the majority of patients [13,14,15]. Additionally, the removal of the primary tumor relieves the mass effect, prevents the release of tumor-associated factors, and allows for pathological assessment of the cancer to inform treatment [16]. In addition to surgery, the evolution of radiotherapy and chemotherapy continued into the late 20th century and remain critical pillars in the treatment of solid cancer today [8]. However, despite the advancements in cancer treatment, recurrence rates have plateaued, due mostly to our inability to effectively eradicate micrometastases [8,10]. Unfortunately, in too many cases, surgery is either too much, such as with Halstedian surgical techniques, or too little, where no amount of surgery would have been curative due to metastatic disease. What the Roman physician Celsus wrote in 100 BC still holds true: “After excision, even when a scar has formed, none the less the disease has returned” [17].

## 2. The Crime: Surgery and Metastasis Formation

**Figure 1:**
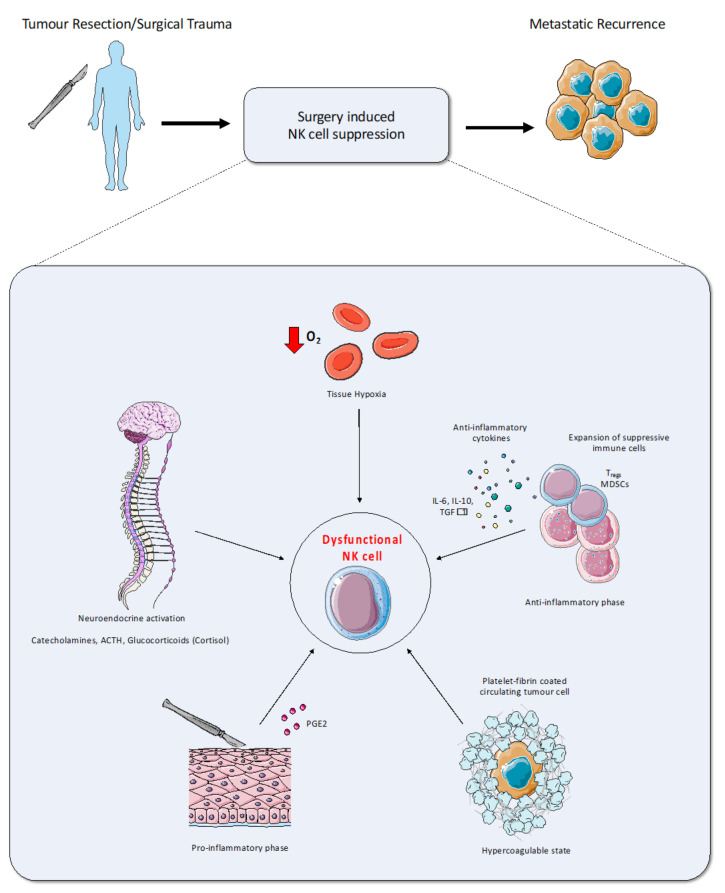
Potential mechanisms of postoperative cancer recurrence. Numerous changes occurring in the postoperative period have been hypothesized to be responsible for NK cell suppression and postoperative metastasis. These include tissue hypoxia, neuroendocrine activation, a pro-inflammatory phase, a hypercoagulable state, and an anti-inflammatory phase characterized by the release of anti-inflammatory cytokines and the expansion of immunosuppression populations in addition to cellular immune suppression.

Paradoxically, in contrast with the curative potential of surgery, surgical resection has long been linked to increased metastases and cancer recurrence [18]. This link was first made more than a century ago in the 1910s when Marie and Clunet found that incomplete excision of implanted tumors resulted in increased rates of metastasis [19]. Similar studies were performed by Tyzzer [20], who ultimately atttibuted this phenomenon to the “athrepsia hypothesis”, whereby tumor proliferation was dependent upon competition for host-derived nutrients [21]. By the mid-20th century, more modern hypotheses on cancer growth and metastasis developed, leading to the concept of the “dormant” cancer cell: cancer cells that remain quiescent while still retaining their ability to proliferate [22]. Various investigators wrote about animal models in which dormant tumor cells could be triggered to grow by some unknown mechanism in response to surgery [18,23]. Over half a century later, the question still remains: What are these mechanisms? Several hypotheses include tissue hypoxia, neuroendocrine activation, hypercoagulation, the initial pro-inflammatory phase, and the subsequent anti-inflammatory phase (shown in Figure 1). Ultimately however, the end result is a negative effect on immune effector cell function, culminating in suppressed cellular immunity. As outlined in the section below, Natural Killer (NK) cells are critical to the anti-tumor response, and thus, their postoperative dysfunction is the prime suspect in the case of pardoxical postoperative metastases [24,25].

## 3. On Trial: Natural Killer Cells as Potent Tumor Killers

Natural Killer (NK) cells, first identified by Kiessling et al., in 1975, are cytotoxic lymphocytes that play a critical role in the innate immune response by destroying circulating stressed, infected, or cancerous cells [26,27,28]. Mature NK cells can be divided into two functional subsets based on the cell surface density of CD56 and the low-affinity Fc-receptor CD16 [29,30]. CD56^dim^CD16^+^ NK cells make up 90% of peripheral blood and splenic NK cells and are preferentially cytotoxic, whereas most lymph node NK cells are CD56^bright^CD16^dim/-^ and readily produce cytokines [29,30]. While NK cells do not undergo clonal selection, they instead express a limited number of germline-encoded receptors [31].

NK cell activity is regulated by the integration of activating and inhibitory ligands through these receptors [31,32]. NK cell activating receptors recognize pathogen-derived antigens as well as stress-induced ligands in what is termed the “induced-self recognition model” [33] and include Natural Cytotoxicity Receptors (NCRs; NKp30, NKp44, NKp46, and NKp80) and the C-type lectin-like receptors NKG2D and CD94-NKG2C [34,35]. In addition to these activating receptors, co-activating receptors, for example DNAX-accessory molecule (DNAM-1, CD226), serve to fine-tune NK cell activity [36,37,38]. These activating signals are antagonized by inhibitory receptors, including Killer Immunoglobulin-like Receptors (KIRs) and C-type lectin-like receptor CD94-NKG2A [30,39], which recognize constitutively expressed self-molecules. Natural Killers must also integrate signals from activating and inhibitory cytokines. IL-2, IL-12, IL-15, and IL-18 [40,41] positively regulate NK cell function, while IL-10 [42,43,44,45,46,47,48,49,50] and IL-6 [51,52,53,54,55] have pleiotropic effects. Transforming growth factor-β (TGFβ) is predominantly anti-inflammatory and is critical for maintaining homeostasis and preventing autoimmunity [56,57].

NK cells mobilized for the immune response induce apoptosis via death receptor ligands such as FasL and TRAIL [58], undergo Ca^2+^-dependent exocytosis of cytolytic granules (perforin and granzymes) [33,34], and secrete numerous cytokines, including interferon-gamma (IFNγ), tumor necrosis factor-alpha (TNFα), and granulocyte monocyte-colony stimulating factor (GM-CSF), which serve to modulate the immune response [33,59]. Finally, NK cells also participate in antibody-dependent cell-mediated cytotoxicity (ADCC) through CD16 [60].

The functional features of NK cells perfectly position them as natural cancer killers with the ability to respond to tumor cells in the absence of immunological priming. For example, numerous induced self-ligands are upregulated during oncogenic transformation, including NKG2D ligands MICA/B and ULBP1-6 [61] and DNAM-1 ligands CD155 and CD112 [62]. Death receptors, including TRAIL-R1/2, are also widely expressed on tumor cells [63,64]. Furthermore, tumor cells downregulate HLA Class I (“self” molecules) in an attempt to escape T cell mediated responses [65], simultaneously releasing the inhibitory “break” on NK cells. By tipping the balance towards activation, NK cells are then able to directly kill cancer cells (via death receptors and cytotoxic granules) and to rally other immune cells to do the same (via immunomodulatory cytokines).

Importantly, circulating NK cell function is correlated with clinical cancer outcomes in both solid and hematological malignancies [66]. In human studies, higher NK cell activity is inversely correlated with cancer risk [67,68,69,70] and reduced NK cell activity [71,72,73,74] is associated with a worse cancer prognosis in numerous cancer types. An 11-year prospective cohort study among 154 Japanese patients assessed the association between PBMC cytotoxic activity at baseline and cancer incidence. They found that both men and women with medium and high cytotoxicity activity had a reduced cancer risk, while low cytotoxic activity was associated with an increased cancer risk, suggesting a role for immune cell cytotoxicity in the prevention of cancer [68]. In patients with colorectal cancer (CRC), an increased number of NK cells was an independent predictor of improved survival [75]. Additionally, several studies have reported that high levels of NK cell activating receptors (CD56, CD57, NKp30, NKG2D, and NKp46) as well as granzyme B were significantly correlated with better overall survival (OS) in patients with a variety of solid malignancies [25,67,71,76,77,78,79,80]. Inhibition of NK cell-dervied IFNγ was associated with higher stage gastric cancers and worse clinicopathological parameters including tumor size, depth of invasion, and lymph node metastasis [81]. In murine metastasis models, NK cell depletion and genetic deficiency of IFNγ or perforin results in increased metastasis [66]. Taken together, this suggests that immune cell, and specifically NK cell, function is critical for the prevention of tumorigenesis.

Tumor cells can employ a plethora of mechanisms to induce NK cell dysfunction, including upregulation of immune checkpoint expression, such as PD-1, prolonged exposure to MHC class 1-deficient tumor cells and/or NK cell activating receptor antagonists [82,83], the secretion of tumor cell-derived exosomes [84], as well as the secretion of inhibitory tumor-derived cytokines, such as IL-10 and TGFβ1 [66,85,86]. In addition, tumor cells employ a variety of mechanisms to down-modulate surface activating receptors such as NKG2D [83]. This leads to alterations in the homeostatic balance of these receptors resulting in dysfunctional NK cell cytotoxicity and cytokine secretion. Thus, despite their innate ability to recognize and target tumor cells, NK cell function can be suppressed by tumor cells.

## 4. Making a Case for Natural Killer Cell Suppression as the Underlying Driver of Postoperative Metastasis

Cellular immune suppression is a universal response to the pathways that are activated in response to surgery. Surgical stress has been shown to cause a decrease in circulating dendritic cells (DCs), and significant dysfunction in T cells and NK cells [24,25,87,88,89,90,91,92]. As outlined above, NK cells play a central role in the early formation and erradication of metastases. For this reason, postoperative NK cell suppression has been investigated as the mechanism responsible for cancer recurrence post-curative surgery.

The literature presents significant evidence to incriminate NK cell dysfunction in the early formation of postoperative metastases. Numerous investigators have established a link between the suppression of NK cell cytotoxicity in the postoperative period and increased metastatic formation in animal models [24,93,94]. Our lab has developed a reproducible mouse model of surgical stress [24,95], whereby DX5^+^ splenic NK cells were isolated from surgically stressed and naïve mice and adoptively transferred into pharmacologically NK cell-depleted tumor-challenged mice. Mice that had received surgically stressed NK cells had significantly increased lung tumor burden compared to mice that had received NK cells from controls [24]. Moreover, adoptive transfer of non-NK immune cells in a similar manner resulted in no signifincant difference in tumor burden between the two groups, highlighting the specific role of surgical stress in impacting NK cell function to induce metastasis. Moreover, animals receiving anesthesia (0.05 mg/kg buprenorphine) alone had similar metastatic burden to control mice, suggesting that the pro-metastatic effect seen postoperatively is anesthesia/analgesia-independent [24,95]. These experiments established NK cells as the critical effector cells responsible for mediating postoperative metastasis.

In human studies, reduced NK cell activity is associated with increased rates of cancer recurrence and death [96,97,98]. Iannone et al., studied NK cells in pancreatic cancer surgery patients and found a significant decrease in cytotoxicity on POD7, which was restored by POD30 [89]. Velásquez et al., also reported that NK cell *in vitro* killing of K562 leukemia cells was suppressed up to five days after surgery in patients with primary bone cancer [90]. We have recently shown that postoperative NK cells from CRC surgery patients have suppressed *in vitro* cytotoxic killing of tumor cells (K562 leukemic cells), which was most profoundly reduced on POD1 and returned to baseline levels by POD28. Postoperative samples also had reduced IFNγ production in response to cytokine stimulation using NKVue™ [25]. The most profound suppression was observed on POD1 where we saw 90.2% (37/41) of patients had IFNγ levels below the minimum detectable level (15.6 pg/mL). As compared to baseline, the mean reduction in IFNγ production was 83.1% (s.d. 25.2%; CI: 75–91). Astoundingly, this suppression persisted until POD28 in 65.5% (19/29) of patients and POD56 in 33.3% (4/12) of patients [25]. Additionally, Reinhardt et al., investigated IFNγ production in response to *Staphylococcus aureus* or IL-12 stimulation in the CD56^Bright^ population of surgery patients and found a significant decrease in IL-12R (CD212) expression and an impairment in IFNγ production on POD1 up to 7 days postoperatively [91].

## 5. The Evidence: Understanding the Mechanisms of Postoperative Natural Killer Cell Suppression

There is strong evidence in murine models and in cancer patients that NK cell dysfunction is responsible for the development of postoperative metastases. The cause (s) of NK cell suppression is currently unknown, although there are numerous hypothesized mechanisms of postoperative metastases, many of which directly or indirectly contribute to NK cell suppression. These mechanisms are discussed below.

### 5.1. Exhibit A: Physiologic Responses to Surgical Stress

#### 5.1.1. Tissue Hypoxia Directly Impairs NK Cell Cytotoxicity while Tumor Cells Thrive

Hypoxia describes a state of reduced oxygen supply and is a common and persistent consequence of surgery [99,100,101,102]. Hypoxia can directly impair NK cell function via Hypoxia-Inducible Factor (HIF) 1α pathway activation, which leads to alteration of the transcriptome, modification of metabolism gene expression, proinflammatory cytokine and chemokine secretion, as well as an impairment in the release of IFNγ, TNFα, GM-CSF, and CCL3 [103,104]. Balsamo et al., cultured NK cells isolated from healthy donor PBMCs under hypoxic (1% oxygen) or normoxic (20% oxygen) conditions. They reported that hypoxia caused a downregulation of surface markers NKp46, NKp30, NKp44, and NKG2D, independent of the presence of IL-2, IL-15, IL-12, or IL-21. Hypoxic NK cells also exhibited lower cytotoxicity against FO-1 melanoma cells due to impaired degranulation [105]. Recent investigations have revealed that hypoxia in NK cells induces the activity of protein tyrosine phosphatase SHP-1 (Src homology region 2 domain-containing phosphatase-1), which attentuates STAT3 and ERK signalling leading to an impairment of NK cell function [106]. Moreover, tumor cells have evolved to use hypoxic stress to their advantage through HIF activation [107,108], resulting in immune suppression and tolerance to immune surveillance by promoting MDSC accumulation, inhibiting DC maturation, and recruiting T_regs_, which ultimately impair NK cell function [104,108,109]. Through these mechanisms of NK cell impairment, hypoxia as a result of surgery and in the context of cancer may therefore play a critical role in postoperative metastases and cancer recurrence [110,111].

Interestingly, despite studies reporting NK cell impairment in response to cytokine stimulation in the context of hypoxia, pre-activation of NK cells with IL-2 was able to abrogate this functional suppression. Importantly, pre-activated NK cells maintained NKG2D expression and could mediate cytoxic killing of multiple myeloma (MM) cells, even under hypoxic conditions [112]. Solocinski et al., demonstrated that genetically-engineered “high-affinity” NK cells (haNKs), which express a high affinity CD16 receptor and endogenous IL-2, were resistant to hypoxia-induced functional suppression. Compared to normal NK cells, which exhibited reduced cytotoxity against PC3, MCF-7, and H460 cell lines, haNK cells maintained high cytoxicity against target cells under hypoxic conditions [113]. Taken together, preoperative adminstration of IL-2 could be a viable therapeutic strategy to prevent postoperative hypoxia-induced functional suppression of NK cells (Table 1). However, this has not yet been explored in the context of a clinical trial with potential adverse effects including increased risk of hypercytokinemia and systemic inflammation [114,115].

#### 5.1.2. Hypothalamic-Pituitary-Adrenal Axis Activation Alters NK Cell Epigenetics

Initially described in response to injury or trauma, the “stress response” is a series of complex physiological events that include neuroendocrine and hypothalamic-pituitary-adrenal (HPA) axis activation [117,118]. The subsequent sympathetic autonomic nervous system activation then results in the release of catecholamines from the adrenal medulla and norepinephrine from presynaptic nerve terminals leading to the activation of adrenergic receptors [118]. In addition, corticotrophin (ACTH) released by the pituitary stimulates the adrenal cortex to release glucocorticoids (GCs), including the stress hormone cortisol, into the bloodstream. Surgery is one of the most potent activators of ACTH/GCs and inhibits the physiologic negative feedback mechanism, resulting in increasing plasma levels of these hormones [117]. HPA activation has complex metabolic effects that are evolutionarily essential for survival post-trauma, although it has been argued that this response to sterile surgery is unnecessary.

Chronic activation of the sympathetic system through stress or pharmological means has been shown to reduce NK cell activty [119,120]. In a rat model of MADB106 mammary adenocarcinoma lung metastasis, psychological stress or adminsitration of metaproterenol, a non-selective β-adrenergic agonist, was shown to reduce NK cell activity resulting in increased metastatic burden [121,122]. β-adrenergic receptor activation resulted in fewer NK cells in the lungs and reduced NK cell cytoxicity against MADB106 cells *in vitro* [117,123]. GCs are also considred to be anti-inflammatory, as they can inhibit the expression of proinflammatory cytokines, such as IL-6, TNF, IL-1β,or IL-12, from monocytes, macrophages, and DCs [124]. Furthermore, GCs have also long been known to inhibit NK cell activity. GCs were shown to alter the expression of surface receptors critical for NK cell functions, including CD16, DNAM-1, NKp46 and NKp30 [125,126]. Treatment of isolated NK cells with the synthetic GC dexamethosone was shown to inhibit mTORC1 leading to reduced IFNγ production following IL-2/IL-12 sitmulation [126]. In addition, GCs have been shown to impair NK cell cytoxiticty as evidenced by reduced perforin and granzyme A/B release [124,127,128]. These immunosuppressive effects are thought to be mediated via epigenetic changes. Treatment of NK cells with dexamethosone was shown to cause a signifcant reduction of H4-K8 acetylation in the IFNγ and perforin promoters. Moreoever, this effect was abrogated by Trichostatin A, a histone deacetylase inhibitor. This suggests that GCs reduce promoter accessibility, thereby reducing expression of NK cell effector molecules. In fact, treatment of NK cells with TSA abrogated GC-induced reduction of IFNγ secretion [128,129].

Current research has focused on targeting adrenergic receptors using β-blockers with promising results showing improved NK cell function along with reduced invasive potential and tumor growth *in vitro* and reduced cancer recurrence or severity in cancer patients [117,118,130] (Table 1). In addition, a Phase 2 randomized trial of perioperative propranolol, a β-blocker, and etolodac in breast cancer patients reported normalization of IL-6 and CRP, decreased EMT, and decreased tumor-infiltrating monocytes [131]. A multi-centre Phase 3 clinical trial to assess immune suppression and cancer recurrence in CRC surgery patients treated with propranolol and etolodac is currently underway (NCT00888767) [132]. Importantly, the cardiopulmonary effects of β-blockers must be considered in the perioperative period. As of yet, there are no therapeutics targeting surgery-induced upregulation of glucocorticoids. However, hypercortosolism can be treated with mifepristone (RU486), the only glucocorticoid receptor (GR) antagonist that is availabe for clinical use [129] (Table 1). In two sutdies, Chen et al., demonstrated that isolated human uterine as well as peripheral blood NK cells showed increased cytoxicity against K562s in the presence of mifepristone, aborgating the immunosuppressive effects of cortisol exposure [133,134]. Morever, mice that underwent LPS-induced immunosuppression had restored humoural and T-cell anti-tumor responses as well as improved tumor clearance when treated with mifepristone (30 mg/kg; i.p.) [135]. Finally, mifepristone signficantly increased the one year surval rate of spontaneous lung cancer in a murine model [136]. Taken together, this illustrates that mifepristone can antagonize glucocorticoid-mediated immune suppression, thereby improving tumor clearance. As a result, administration of mifepristone could be a viable perioperative treatment to address the role of glucocorticoids in mediating postoperative immunosuppresion. Potential side effects of mifepristone use include hypokalemia, hypertension, and adrenal insufficiency [137,138].

#### 5.1.3. The Postoperative Hypercoagulable State Shields Tumor Cells from NK-Mediated Clearance

Surgery and cancer independently have been associated with a hypercoagulable state. This is characterized by increased tissue factor, fibrin, and thrombin, platelet activation, and the formation of clots around tumor cell emboli and circulating tumor cells (CTCs) [95,139]. These clots may facilitate micrometastases, tumor cell adherence to endothelial cells [95] and importantly, immune evasion. CTCs, which are critical for the formation of distant micrometastases, can be coated by platelets and firbin, resulting in physical protection from NK cells in the blood stream [140]. Nieswandt et al., demonstrated that the presence of platelets protected Yac1 cells from NK cell cytotoxicity *in vitro*. Blocking platelet aggregation using hirudin, heparin, ADP scavenger apyrase, and anti-P-selectin mAbs reversed the platelet-mediated protection by inhibiting platelet aggregation [141]. This platelet coating can also secrete TGFβ, which inhibits NK cell cytoxicity by impairing granule mobilization and IFNγ secretion [142]. Placke et al., reported that co-culture of MHC I negative NCCIT cancer cells with a thousandfold excess of platelets led to membrane protein transfer resulting in the expression of platelet-derived MHC I molecules on the cancer cells. Consequently, this acquired “pseudoself” phenotype countered the “missing self” mechanisms critical to NK cell anti-tumor cytotoxicity, leading to reduced killing of cancer cells compared to controls [143,144]. Taken together, there are numerous mechanisms by which a hypercoagulable state resulting in platelet-fibrin clots may subvert NK cell-mediated tumor cell clearance.

Interestingly in murine studies, platelet depletion and P-selectin deficiency were shown to reduce experimental metastases and fibrinogen-deficient mice demonstrated significantly reduced implanted and spontaneous metastatic foci [145,146,147,148,149,150]. Clinically, perioperative anticoagulation has been associated with increased cancer-specific survival in cancer surgery patients [151,152,153]. Seth et al., investigated the role of surgery-induced hypercoagulation on metastases in a murine model and found that treatment with low molecular weight heparin (LMWH) was able to reduce metastases through the inhibition of peritumoral clot formation [95] (Table 1). Furthermore, this effect was NK cell-mediated in that LMWH was unable to attenuate metastasis in NK cell-depleted surgically stressed mice [95]. The ability of LMWH to reduce metastases and increase survival has been documented in both murine models and patients with solid malignancies [151,152,154,155,156]. For these reasons, our group is conducting a Phase 3 clinical trial in which perioperative LMWH is given to CRC surgery patients in an effort to reduce postoperative metastasis (PERIOP-01; NCT01455831) [157,158]. Regardless of the context, the use of LMWH carriers the risk of thrombocytopenia and internal bleeding [159,160].

### 5.2. Exhibit B: Immune-Mediated Responses to Surgical Stress

#### 5.2.1. Pro-Inflammatory Prostaglandins Increase Tumorgenicity and Suppress NK Cell Function

Despite advancements in surgical technique, injury to healthy tissue is unavoidable, resulting in an acute inflammatory response mediated by soluble factors [110]. While necessary for wound healing, these soluble factors also stimulate cancer cell proliferation and migration. When combined with disrupted endothelium, this constitutes an ideal condition for CTC seeding after surgery [161]. One such factor is the COX-2 product prostaglandin E2 (PGE2), which can be both released by tumor cells [130] and act directly on tumor cells to induce metastatic activity, proliferation, adhesion, migration, extracellular matrix invasion, resistance to apoptosis, and the secretion of proangiogenic factors [130]. Interestingly, in cancer patients, PGE2 is associated with increased tumor size and stage, recurrence, and decreased OS [130]. In the postoperative period, PGE2 was shown to be elevated from hours 9 through 30 postoperatively in the CSF and at the surgical site of osteoarthritis patients undergoing primary total hip arthroplasty [162]. Furthermore, using a rat tumor metastasis model, Yakar et al., reported that exogenous PGE2 resulted in a dose-dependent increase in MADB106 lung tumor retention and dose-dependent suppression of NK cell activity. Additionally, selective depletion of NK cells abrogated PGE2-mediated lung tumor retention [163], suggesting a role for PGE2-dependent suppression of NK cells and postoperative metastasis. In fact, PGE2 is known to suppress NK cell effector functions directly through four endogenous PGE2 receptors, EP1–4 [164,165], and indirectly via the downregulation of the common γ chain receptor subunit [166].

In terms of potential therapeutics, COX-2 inhibitors (i.e., non-steroidal anti-inflammatory drugs (NSAIDs)) prevent the synthesis of prostaglandins and have been studied as long-term chemopreventers of malignancy due to their ability to increase tumor cell apoptosis, decrease proangiogenic agents, and reduce tumor microvascular density [167,168,169] (Table 1). However, NSAIDs have also been shown to suppress NK cell cytokine secretion in a COX-independent manner [170] and are therefore unlikely to be of use to prevent NK cell suppression in the postoperative period. The small molecule inhibitor RQ-15986 has been shown to block EP4-mediated NK cell suppression and inhibit growth of implanted tumor cells and lung metastases in a murine model of breast cancer *in vivo* [165]. Thus, RQ-15986 may prove to be a viable therapeutic to combat surgery-induced NK cell suppression and metastasis.

#### 5.2.2. The Compensatory Anti-Inflammatory Phase as the Scene of the Crime

The prolonged postoperative anti-inflammatory phase was first described by Bone et al., in 1997 as “compensatory anti-inflammatory response syndrome” (CARS) in the context of sepsis [171]. They described a compensatory reaction that could be as great or greater than the initial pro-inflammatory response, the purpose of which was to restore homeostasis [171]. It is now understood that there are overlapping concurrent pro- and anti-inflammatory responses throughout the postoperative period. The extent of surgical trauma is reflected in the degree of inflammatory cytokine production, which in turn has been shown to determine the degree and duration of the subsequent anti-inflammatory sequelae [172]. Hence, the evolutionary motive for postoperative NK cell dysfunction is the achievement of homeostasis. Thus, it may be necessary to mediate both pro- and anti-inflammatory postoperative reponses. The anti-inflammatory phase is characterized by the release of anti-inflammatory cytokines as well as the expansion of immunosuppressive populations.

## 6. Increased Secretion of Inhibitory Soluble Factors: Hostile Witnesses?

### 6.1. Interleukin-6

IL-6 was initially identified as a B cell stimulatory factor and is produced by monocytes, macrophages, and T cells, as well as non-immune cells [55]. It plays a critical role in inflammation by promoting plasma cell differentiation and antibody production [173], inhibiting regulatory T cell (T_reg_) formation, inducing the differentiation of helper T (T_h_) 17 cells [174], and promoting cytotoxic T lymphocyte (CTL) differentiation [55]. The IL-6 receptor consists of two subunits: IL-6Rα (gp80, CD126) and IL-6Rβ (gp130, CD130) and signaling can occur via classical signaling (initiated by ligation with membrane-bound IL-6Rα) or trans-signaling via soluble IL-6Rα [174]. Regardless of initial pathway activation, IL-6 signal transduction occurs via the activation of many intracellular pathways including: Jak1/TYK2 and downstream STAT1, 3, and 5, the PI3K/Akt pathway, the MAPK pathway, and the MEK-extracellular receptor kinase (ERK) 5 pathway [55,174,175].

IL-6 is known to induce the “acute phase response”, characterized by the production of liver proteins such as C-reactive protein (CRP), α_2_ microglobulin, and other proteinases. Postoperatively, IL-6 increases within 30–60 min of tissue damage, peaks at 24 h, and remains high for 72 h post-trauma. In addition, the magnitude of IL-6 production reflects the degree of tissue damage [117,176,177]. Narita et al., compared postoperative serum cytokine levels in patients with prostate cancer undergoing laparoscopic (*n* = 66) vs. open radical prostatectomy (*n* = 99). They found that IL-6 and CRP levels increased immediately postoepratively in both groups, but were significantly lower in the laparoscopic group on POD1. Clinicaly, postoperative IL-6 and CRP are known to be useful markers of postoperative morbidity, including infection, in cancer and non-cancer patients. In 1992, Oka et al., first reported a relationship between postoperative serum IL-6 levels >400 pg/mL and the incidence of postoperative complications in cancer surgery patients [178]. These findings of increased postoperative complications in both cancer and non-cancer surgery patients have been supported by numerous other studies to date [179,180,181], which is why IL-6 is often used as an indicator of surgical stress. IL-6 also serves as the switch between pro- and anti-inflammatory phases by inducing anti-inflammatory factors including glucocorticoids, soluble TNFα receptors, PGE2-dependent IL-10, arginase-1 (ARG1) expression in MDSCs, and TGFβ1 [172,182]. The dual pro- and anti-inflammatory effects of IL-6 are thought to be partially mediated by differences in classical signalling versus trans-signalling [176,183,184].

Although there is a paucity of research into the effects of IL-6 on NK cell function, IL-6 is reported to be a potent inhibitor of NK cell cytotoxicity. Cifaldi et al., reported reduced expression of perforin and granzyme B in human peripheral NK cells exposed to rIL-6 [52]. Furthermore, the addition of soluble IL-6R or the IL-6R mAb tocilizumab *in vitro* restored perforin and granzyme B expression [52]. NK cell cytotoxicity was reduced in patients with heart failure and this corelated with increased levels of IL-6 produced by unstimulated PBMCs [53]. Kang and colleagues examined NK cell function in the context of endometriosis and found that IL-6 in peritoneal fluid was able to suppress NK cell differentiation and cytotoxicity via the adapter protein tyrosine phosphatase SHP-2 [54,55]. In the context of cancer, Scheid et al., showed that NK cells isolated from 20 patients with advanced colon and pancreatic cancer had a 50% reduction in cytotoxicity on day 7 after treatment with rIL-6, which was reproduced with an additional exposure to rIL-6 at day 22 [185]. Furthermore, increased levels of IL-6 were correlated with poorer prognosis in many cancer types, including breast cancer, prostate cancer, metastatic CRC, MM, and non-small cell lung cancer (NSCL] [184,186,187,188,189,190]. Taken together, these studies suggest an inhibitory role for IL-6 in the context of NK cell function.

In the context of metastatic cancer, there are currently over 200 clinical trials investigating the safety and efficacy of various therapeutics targeting the IL-6 pathway, including IL-6 direct inhibitors (i.e., Siltuximab), IL-6Rα direct inhibitors (i.e., Tocilizumab), IL-6R gp130 direct inhibitors (i.e., Raloxifene), and JAK inhibitors (i.e., Ruxolitinib) (Clinical Trials Registry, www.clinicaltrials.gov, accessed date: 21 May 2021) (Table 1). Studies to date show mixed results with the majority of therapeutics being well-tolerated but ineffective [184,191,192]. This is perhaps due to the dual pro- and anti-inflammatory effects of IL-6 [176,183,184]. Specifically, siltuximab monotherapy has been shown to be well tolerated with no improvement in clinical outcomes in patients with ovarian, pancreatic, CRC, head and neck, and prostate cancers, NSCLC, MM, and renal cell carcinoma [193,194,195,196,197]. In the context of surgery, IL-6 has been shown to play an important role in wound healing [198,199]. Hence, anti-IL-6 therapeutics may impair postoperative healing. However, one study by Locci et al., assessed postoperative wound healing and complications in patients with rheumatoid arthritis taking Tocilizumab (IL-6Rα direct inhibitor). The mean delay between Tocilizumab therapy and surgery was 4.94 weeks with 38 patients stopping Tocilizumab less than 4 weeks before surgery. They found that only 8.9% of patients experienced postoperative complications and concluded that Tocilizumab was not associated with increased rates of postoperative complications [200]. Thus, while provocative, blocking IL-6 might have unintended negative consequences in the context of oncologic surgery, suggesting that it may be more appropriate to target postoeprative NK cell function directly.

### 6.2. Interleukin-10

IL-10 is a critical immunoregulatory cytokine produced by T cells, B cells, NK cells, macrophages, and DCs [57]. Historically, it has been described both as “cytokine synthesis inhibitory factor” (CSIF) due to its role as an inhibitor of T_h_1 activation and cytokine secretion [57], and as “B cell-derived T cell growth factor” (B-TCGF) due to its ability to stimulate CD8^+^ T cells [201]. IL-10 has since been reported to also inhibit macrophage cytokine and chemokine secretion, CTL CD28 expression and cytokine production, and T_h_1 proliferation as well as to antagonize DC activity [43,49]. IL-10 is thus essential for the prevention of autoimmunity.

While still widely regarded as a predominantly anti-inflammatory cytokine, IL-10 has recently been shown to have pleiotropic effects on NK cells: inducing *in vitro* NK cell cytotoxicity [42,43,44,45,46,47,48] while inhibiting cytokine production, including IFNγ and TNFα [49,50]. The IL-10 receptor is composed of two subunits: IL-10R1 and IL-10R2. In NK cells, IL-10 ligation with its receptor has been shown to activate JAK1 and non-receptor tyrosine kinase 2 (TYK2), resulting in the phosphorylation and activation of STAT1, 3, and 5 [57]. The exact pathways by which IL-10 exerts activating versus inhibitory effects on NK cells require further investigation and may be influenced by the activity of other immune cells, as well as the cytokine milieu.

IL-10 has also been shown to increase significantly following surgical stress [202,203,204]. In the context of major abdominal surgery, both IL-10 mRNA and serum IL-10 were shown to be increased on POD1 [203]. Kato and colleagues reported that IL-10 achieved a maximum value four hours after skin incision with levels returning to baseline by POD1 [203]. In 11 infants undergoing cardiopulmonary bypass operations, IL-10 levels also peaked 24 h after termination of bypass (351.0 +/− 304.0 pg/mL) [204]. In patients with cancer this increase in IL-10 could be detremental for patients undergoing surgery by contributing to postoperative NK cell suppression.

In the context of cancer, pegylated IL-10 has been shown to mediate tumor regression via tumor-infiltrating CD8^+^ T cell expansion and enhacement of immune checkpoint inihibitors [205]. Conversely, a meta-analysis of serum IL-10 in 1788 cancer patients showed that high seurm IL-10 levels were significantly associated with worse OS and disease-free survival (DFS) at 1 year, 3 years, and 5 years for both solid and hematological malignancies [206]. In addition, *in vitro* rIL-10 has been shown to act as a tumor growth factor to enhance human melanoma cell proliferation [205]. Thus, IL-10 may not be an ideal target given its pleiotropic effects on NK cells and in the context of cancer. A potential therapeutic may include the use of an anti-IL-10 monoclonal antibody, such as BT063 [207], to block the suppressive effects on postoperative NK cells, although there is currently a paucity of studies describing the clinical use of such a therapeutic (Table 1).

## 7. Transforming Growth Factor β1

TGFβ1 is essential to wound healing. During granulation tissue formation, TGFβ1 induces the expression of fibronectin, collagen I and III, and VEGF in addition to improving the angiogenic properties of endothelial progenitor cells, promoting keratinocyte migration, and stimulating contraction of fibroblasts [208]. TGFβ1 may therefore be important in postoperative wound healing in response to surgical trauma, however there is a paucity of research investigating TGFβ1 in the postoperative period. Our group profiled 26 cytokines and chemokines using a multianalyte protein array in B16LacZ tumor-bearing surgically stressed and untreated mice [24]. At 18 h post-operation, surgically stressed mice showed a significant increase in plasma TGFβ1, IL-5, and IL-6 [24]. While there exists a knowledge-gap when it comes to the postoperative period, TGFβ1 is well known to have pro-tumorigenic and anti-inflammatory properties. In fact, TGFβ1 is pathologically upregulated in humans as a result of tumor cell proliferation, can be secreted by tumor cells, and is a negative predictor of DFS and OS [116,209,210,211]. Moreover, there is a direct relationship between TGFβ1 levels and metastatic burden in a variety of cancers [210,212,213]. Thus, TGFβ1 may therefore be a provocative target to reverse postoperative immune suppression and prevent cancer recurrence.

In terms of its immunosuppressive properties, TGFβ1 has been shown to promote the development of T_regs_ and suppressive myeloid-derived suppressor cells (MDSCs), prevent the activation and differentiation of CD4^+^ and CD8^+^ T cells, inhibit the maturation and antigen presenting capacity of DCs via downregulation of MHC II, and dampen NK cell proliferation, cytotoxicity, and IFNγ secretion [56,77,213,214]. In culture, TGFβ1 can override IL-2 activation of NK cells and induce the downregulation of NKp30, NKp46, DNAM-1, and NKG2D [214,215]. In addition to the downregulation of NK cell receptors, TGFβ1 is reported to suppress IFNγ production through Smad-dependent repression of “master regulator” transcription factor T-bet [216]. Moreover, Viel et al., showed that TGFβ1 signaling opposed the IL-15-induced phosphorylation of both S6 and 4EBP1 (mTORC1 substrates) and Akt (mTORC2 substrate). The effect of TGFβ1 was comparable to the mTORC1 inhibitor rapamycin [56], suggesting a critical role for mTOR inhibition in mediating the effects of TGFβ1 on NK cells. Furthermore, suppression of TGFβ1 signaling in NK cells reduced metastases in two murine models of cancer (B16-F10 melanoma and RM-1 prostate adenocarcinoma) [56]. TGFβ1 was also able to reduce NK cell production of perforin, granzyme B, IFNγ, and macrophage inflammatory protein (MIP)-1β in response to stimulation by K562s in the presence of IL-2. In vivo, NK cell development was arrested by TGFβ1 signaling or mTOR depletion while mTOR activity and NK cell cytotoxicity were enhanced by TGFβRII depletion [56].

Potential therapies targeting postoperative TGFβ1 can be divided into three categories: (1) preventing ligand-receptor interactions using ligand traps (soluble receptors and mAbs, i.e., fresolimumab) [217,218], (2) anti-receptor mAbs (i.e., LY3022859 [219]), and (3) preventing TGFβ1 signal transduction (i.e., BBI608 [220] or Celecoxib [221]) (Table 1). Viel et al., showed that the addition of an anti-TGFβ mAb enhanced IL-15-induced mTOR activity in murine splenocytes [56]. Mariathasan and colleagues showed that co-administration of anti-TGFβ and anti-PD-L1 mAbs in an EMT6 murine model of mammary carcinoma facilitated T cell-mediated anti-tumor immunity and tumor regression [222]. Nam et al., assessed the efficacy of an anti-TGFβ mAb in a transplantable 4T1 model of metastatic breast cancer and reported increased infiltration of T and NK cells, increased expression of NKG2D and cytotoxic granules, and enhanced CD8^+^ T cell-mediated anti-tumor responses [223]. Alvarez and colleagues also investigated the effects of an anti-TGFβ mAb in combination with rIL-2 and found significant increases in the numbers and functionality of NK cells and CD8^+^ T cells. In addition, this combination therapy significantly increased survival of mice injected with 3LL Lewis lung carcinoma cells in a CD8^+^ T cell- and NK cell-dependent manner. Despite these promising results in preclinical cancer models, targeting TGFβ1 in the postoperative period may have adverse effects related to wound healing, as TGFβ1 is known to play a role. However, this role is complex as TGFβ1 can both stimulate and protract wound re-epithelialization. One study investigated the temporal effects of TGFβ in a rabbit dermal ulcer model and reported the administration of an anti-TGFβ mAb early after injury resulted in delayed wound healing, while administration of the mAb at 7 days post-injury did not affect wound healing but reduced hypertrophic scar formation [224]. This is likely due to TGFβ-mediated fibrosis [224,225]. Thus, there is more to be learned about the role of TGFβ in postoperative immune suppression to inform whether the above therapeutic strategies would prove to be beneficial in reducing metastasis and cancer recurrence.

## 8. The Expansion of Immunosuppressive Cell Populations: Willing Accomplices?

Two major postoperative suppressive cell populations are T regulatory cells (T_regs_) and myeloid-derived suppressor cells (MDSCs) [176,183].

### 8.1. Regulatory T Cells

Regulatory T cells are a highly immunosuppressive subset of T cells that play an important role in maintaining immune homeostasis. T_regs_ were originally indentified by Sakaguchi et al., as CD4^+^CD25^+^ T cells with crucial roles in maintaining self-tolerance and thus preventing autoimmunity [226,227,228]. T_regs_ are a heterogeneous population that can be divided into: CD45RA^+^FoxP3^low^ resting T_regs_, CD45RA^-^FoxP3^high^ activated T_regs_, and CD45RA^-^FoxP3^low^ cytokine-secreting T_regs_ [229]. Activated T_regs_ can inhibit the maturation of antigen-presenting cells such as DCs in an antigen-specific manner. Moreover, they can impact immune cell function by consumption of IL-2 via the high affinity IL-2 receptor, secretion of inhibitory cytokines including IL-10, TGFβ, and IL-35 and degradation of ATP [230]. Finally, T_reg_ function and proliferation are promoted by catecholamines and prostaglandins [231,232,233,234]. As a result of their role in immune regulation, T_regs_ have been implicated in many diseases, including cancer.

In the context of cancer, T_regs_ have been reported to infiltrate the TME in both murine and human tumors accounting for up to 50% of CD4^+^ T cells. Furthermore, increased infiltration of T_regs_ is associated with poor prognosis [235,236,237,238,239]. Within the TME, T_regs_ can suppress various types of effector lymphocytes inculding CD4^+^ T_h_ cells, CD8^+^ CTLs, and NK cells. For this reason, T_reg_-specific immunotherapies have emerged as a promising therapeutic option for cancer patients [183]. In terms of surgical stress, T_regs_ were reported to decrease immediately following surgery, due to their association with the TME [228]. However, Saito et al., collected blood from cancer patients pre- and postoperatively until POD6 and found that regulatory T cell subsets increased to higher levels than those observed preoperatively and that this increase was proportional to surgical stress and invasiveness of the surgery, revealing T_regs_ to be a novel marker of surgical stress [240]. This was also observed in patients who received a radical mastectomy where the peripheral T_reg_ population was significantly expanded postoperatively [232]. Interestingly, postoperative stress was reported to induce T_regs_ expression of FoxP3, TGFβ1, PD-1 and PD-L1, suggesting that postoperative T_regs_ may have greater suppressive capacity than their preoperative counterparts [241]. Thus, the expansion of T_regs_ in the postoperative period may play a critical role in the maintenance of an anti-inflammatory state, resulting in cellular immune suppression and cancer recurrence.

A growing body of literature has revealed the important suppressive activity of T_regs_ on NK cell functions [242]. As reviewed by Orentas et al., a detectable expansion in the T_reg_ cell subset in many types of cancers was inversely correlated to the frequency and function of NK cells [243]. T_regs_ have been shown to suppress NK cell function via numerous mechanisms, most notably cytokine and soluble factor release [227,244], membrane-bound TGFβ expression [227,245], and competitive IL-2 consumption [244]. Ghiringhelli et al., demonstrated that *in vitro* co-culture of NK cells with T_regs_ led to reduced IL-12-mediated IFNγ secretion and K562 lysis as compared to co-culture with conventional CD4^+^CD25^+^ T cells. In a murine model, NK: T_reg_ co-culture similarly resulted in suppression of Yac1 cell lysis [246]. However, TGFβ^-/-^ T_regs_ were unable to suppress NK cell function, suggesting the importance of TGFβ in T_reg_-mediated immunosuppression [246]. In vivo murine injection of T_regs_ significantly increased B16 metastatic burden compared to control, conventional T cells [246]. Morever, the authors demonstrated that membrane-bound TGFβ mediated contact-dependent mechanisms of suppression leading to reduced NKG2D. This, alongside other studies, demonstrated that blocking this interaction can prevent NK cell inhibition [246]. In addition to the expression of membrane-bound TGFβ, T_regs_ have also been reported to secrete TGFβ as well as IL-10, IL-35, granzyme A/B, and perforin [227]. Finally, T_regs_ express IL-2R while producing very little IL-2, thus resulting in increased IL-2 consumption. IL-2 is critical for both the expansion and maintenance of T_regs_ as well as their suppressive functions [247]. Furthermore, the consumption of IL-2 has been shown to limit IL-2-mediated activiation of NK cells and subsequent IFNγ and granzyme B production and TRAIL expression in mice [227].

Depletion of T_regs_ by genetic knockout or pharmological inihbition has led to improved NK cell activty and tumor clearance in both human and murine studies [246]. This suggests that targeting T_regs_ could be a viable therapuetic strategy in the perioperative period. Several clinically available therapeutics have demonstrated efficacy in selectively targeting T_regs_ and improving NK cell function. Ghiringhelli et al., demonstrated that low oral doses of cyclophosphamide in advanced chemotherapy-resistant cancers provided a selective reduction of T_regs_ while restoring NK cell activity [248] (Table 1). In addition, PD-1 and CTLA-4 checkpoint blockade was shown to reduce T_reg_ activity and improve immune effector function [249,250]. Furthermore, lenalidomide (Revlimid; CC-5013) and pomalidomide (CC-4047) are immunomodulatory drugs that are approved for the treatment of MM and have been shown to inhibit the proliferation and function of T_regs_ [251] (Table 1). Davies et al., reported an expansion of CD3^-^CD56^+^ NK cells with significantly higher cytotoxicity against MM cell lines in MM patients who had received lenalidomide or pomalidomide, as compared to controls. Finally, depletion of CD56^+^ cells blocked the drug-induced MM cell lysis, further suggesting that the anti-tumor effects of these drugs are mediated via NK cells [252]. Potential side effects of these therapeutics include anemia, impaired wound healing, thrombocytopenia, and deep vein thrombosis.

The suppressive effects of T_regs_ have also been blocked by the presence of IL-2, IL-4, IL-7, and IL-12 *in vivo* in cancer patients [253]. This suggests that cytokine administration prior to surgery could provide a protective effect against T_reg_-mediated immunosuppression. Moreover, NK cell activity is inversely proportional to T_reg_ counts as observed by Chin et al., implicating that the activation of either cell type inhibits the other [254]. Brillard et al., reported that autologous IL-2-activated NK cells in humans and mice blocked T_reg_ proliferation via the secretion of high levels of IFNγ, skewing the environment towards T_h_1 polarization [255]. Furthermore, Roy et al., using *Mycobacterium tuberculosis* as a model, demonstrated that NK cells inhibited the conversion of FOXP3^+^ cells into T_regs_ and induced direct apoptosis of existing T_regs_ during the response to infection in healthy individuals [256]. Thus, given T_regs_ ability to potently suppress NK cell function and the context of their postoperative expansion, therapeutics to deplete postoperative T_regs_ or boost NK cell function to overcome T_reg_-mediated suppression should be further investigated for perioperative use.

### 8.2. Myeloid-Derived Suppressor Cells

Evolutionarily, myeloid cells are important host protectors, acting to prevent infection and aid in tissue remodelling [257]. However, chronic inflammation, infection, and cancer result in persistent myelopoiesis that generates myeloid cells with aberrant genomic profiles and suppressive activity [257,258]. These suppressive cells are termed myeloid-derived suppressor cells and are characterized as a heterogenous population of immature myeloid lineage immunoregulatory cells. MDSCs are hypothesized to develop via a two-signal model. The first signal serves to inhibit terminal differentiation of myeloid progenitors and the second signal induces their pathological activation [257]. This signal is produced in response to chronic inflammation and includes stimulation with GM-CSF, G-CSF, M-CSF, VEGF, and polyunsaturated fatty acids. The second signal is mediated by pro-inflammatory cytokines and DAMPs and includes stimulation with IFNγ, IL-1β, IL-4, IL-6, IL-13, TNF, and HMGB1 [259]. MDSCs are made up of granulocytic or polymorphonuclear- MDSCs (PMN-MDSCs) and monocytic-MDSCs (M-MDSCs), with M-MDSCs being more suppressive on a per cell basis. PMN-MDSCs are phenotypically and morphologically similar to neutrophils, while M-MDSCs are similar to monocytes. Perhaps due to their relatively recent coming of age in the immunology world, there is still some controversy around how to define these cells, what their specific functions are, and the mechanisms by which they accomplish them. Phenotypically, these cells are lineage marker negative (CD3^-^, CD56^-^, and CD19^-^). Human PMN-MDSCs are generally identified as CD11b^+^CD14^-^CD15^+^CD33^+^ and M-MDSCs are identified as CD11b^+^CD14^+^CD15^-^CD33^+^HLA-DR^-/lo^ [259]. Morphologically, MDSCs display weak phagocytic activity, increased reactive oxygen species (ROS) formation, high expression of ARG1, inducible nitric oxide synthase (iNOS), COX-2, and anti-inflammatory cytokines TGFβ and IL-10 [257,259,260]. Regardless of their nomenclature, MDSCs are ultimately defined by their ability to suppress immune cell function.

Cancer results in chronic inflammation and many of the “first signals” in the two-signal model of MDSC development are produced by tumor cells [258]. As such, MDSCs play a critical role in mediating tumorigenesis and immune evasion. MDSCs can directly promote tumor progression by affecting TME remodelling and angiogenesis via soluble factors like VEGF and can inhibit tumor cell senescence by antagonizing IL-1α [257,260]. Moreover, MDSCs induce immune cell tolerance through immune cell supression through the various mechanisms described above. Clinically, peripheral MDSCs are an independent indicator of poor prognosis and poor outcome in solid and hematological malignancies and can help predict response to cancer therapies [259,261]. Veglia et al., recently summarized pre-clinical and clinical studies investigating the role of MDSCs in cancer [259]. A positive correlation between circulating MDSCs and cancer stage/tumor burden has been reported in colorectal carcinomas, NSCLC, breast, bladder, and thyroid cancers [261,262,263,264,265,266,267,268]. Zhang et al., performed a systematic review assessing the relationship between MDSCs and the prognosis of patients with solid tumors and reported elevated circulating MDSCs were an independent indicator of poor patient outcomes [269]. This is corroborated by studies that have shown shorter progression free interval/OS in patients with NSCLS, CRC, bladder, thyroid, uterine, or cervical cancer [261,266,267,268,270,271,272]. In hematological malignancies, M-MDSC numbers correlated with reduced survival in patients with MM and lymphoma (Hodgkin’s, non-Hodgkin’s, diffuse late B cell) [273,274,275]. In fact, in 2016, MDSCs were shown to predict resistance to checkpoint inhibitors (CPIs) [276]. Thus, the presence of MDSCs is detrimental for cancer patients and provides a complex target for cancer immunotherapies.

Acute physiologic insult results in the recruitment of granulocytes and the release of endogenous danger signals and inflammatory mediators into the circulation. In response, hematopoietic stem and progenitor cells in the bone marrow undergo a process termed “emergency myelopoiesis”, which results in the production of myeloid cells, including MDSCs. It is well established that inflammatory mediators such as IL-1, IL-6, and prostaglandins stimulate this accumulation of MDSCs [277,278]. Sander et al., showed that MDSC accumulation was dependent upon gp130 (IL-6) signaling, as gp130-deficient mice did not accumulate MDSCs following sepsis [279]. As discussed, IL-6 and prostaglandins are highly upregulated in response to surgical stress, implicating a role for surgery as a driver of emergency myelopoiesis [280,281]. Although this process is critical for potentiating early innate immune responses, it also contributes to the expansion of highly immunosuppressive cells, which provide an immunological window for tumor cell survival following surgery. MDSCs have recently been shown to expand rapidly in response to surgical stress in both murine models [282,283,284] and in humans [84,285,286]. In a 4T1 breast cancer model, Ma et al., showed a postoperative increase in MDSCs that preferentially infiltrated the TME and promoted metastasis. MDSCs promoted EMT of tumor cells through TGFβ, VEGF, and IL-10. In addition, anti-Gr1 antibody treatment reduced postoperative pulmonary metastases [283]. Similarly, Xu et al., showed that surgery results in an increase in MDSCs and a concomitant increase in colorectal cancer CT26 tumor cell growth via chemokine (C-X-C motif) ligand 4 (CXCL4) downregulation. Inoculation using a CXCL4 over-expressing CT26 tumor abrogated MDSC infiltration and reduced MDSC migration *in vitro* [284]. In addition, Wang et al., demonstrated a significant increase in M-MDSCs in lung cancer patients after thoracotomy as compared to preoperative levels. Furthermore, M-MDSC expansion positively corelated with T_reg_ expansion [285].

As previously stated, MDSCs are ultimately defined by their ability to suppress both innate and adaptive immune cell function. In terms of Natural Killer cells, MDSCs are able to suppress NK cell functions through both contact-dependent and -independent mechanisms, as previously reviewed by Market et al. [183]. Contact-dependent mechanisms include the engagement of germline receptors, such as TIGIT [287] and NKp30 [288], as well as via the expression of membrane-bound TGFβ1 [289]. Contact-independent mechanisms include the upregulation of ARG1 and/or iNOS and/or and the generation of nitric oxide (NO) [290].

While some mechanisms of MDSC-mediated immune suppression and tumor progression have been elucidated, the mechanism(s) by which surgery-induced MDSCs (sxMDSCs) mediate postoperative cancer recurrence are currently incomplete. sxM-MDSCs were reported to interact in a contact-dependent manner with CD4^+^ T cells to induce the expansion of FoxP3^+^ T_regs_. Additionally, differentiation of MDSCs in the context of a murine model was shown to reduced postoperative sxM-MDSCs and T_regs_ and resulted in fewer lung nodules [285]. Taken together, this strongly implicates sxMDSCs and T_regs_ in the development of postoperative metastasis.

Due to their association with cancer severity and survival and their immunosuppressive capacity, MDSCs have become an important immunotherapeutic target. Specifically, MDSC modulation is associated with significant therapeutic benefit in preclinical models and early phase trials (reviewed in 2018 by Fleming et al.) and has been achieved by: (1) inhibiting MDSC immunosuppressive activity, (2) blocking MDSC migration to the TME, and (3) depletion of MDSCs [260]. The activity of iNOS, ARG1, and COX-2 can be inhibited using phosphodiesterase type 5 (PDE5) inhibitors (sildenafil and tadalafil) [260,282] and the class I histone deacetylase inhibitor entinostat alone or in combination with anti-PD-1 antibodies [260] (Table 1). Our group studied the effects of the PDE5 inhibitor sildenafil in a murine model of surgical stress and found that sildenafil was able to abrogate the suppressive effects of sxMDSCs on NK cells and reduce lung metastases postoperatively [282]. We and others have shown that PDE5 inhibitors can decrease MDSC ARG1 and iNOS expression, and we hypothesize that this is the mechanism responsible for the observed effects *in vivo* [291]. We are currently testing the effects of the PDE5 inhibitor tadalafil in conjunction with an influenza vaccine on MDSC function in a Phase 1b clinical trial (PERIOP-04) [292]. ARG1 activity can also be inhibited by nor-NOHA, which has been shown to improve NK cell ADCC in 4T1 bearing mice [293]. The transcription factor STAT3 is involved in immunosuppressive pathways and can be blocked using siRNA or decoy oligonucleotides in combination with immune checkpoint inhibitors or CpG oligonucleotides (TLR9 agonists). In addition, pharmacological inhibition of fatty acid oxidation has been shown to decrease the immunosuppressive capacity of MDSCs. MDSC migration can be impaired by blocking chemokine signaling using small molecule inhibitors or chemotherapeutic drugs, such as Reparixin [260,294] or MK7123 [260,295]. The MDSC population can be depleted by inhibiting the conversion of immature myeloid cells into MDSCs using dimethyl amiloride or omeprazole to reduce extracellular vesicles in serum. Furthermore, MDSCs can be induced to differentiate into mature DCs or macrophages using the vitamin A derivative all-trans retinoid acid (aTRA) or chemotherapeutic agents like docetaxel. In a murine model, perioperative atRA, known to induce the differentiation of immature cells, reduced postoperative sxM-MDSCs and T_regs_ and resulted in fewer lung nodules [285]. Finally, tyrosine kinase inhibitors like sunitinib have been reported to reduce circulating MDSCs, chemotherapeutics like gemcitabine and 5-fluorouracil can induce selective apoptosis of MDSCs, and mAbs such as anti-Gr1 mAb have been used to deplete MDSCs in murine models [260,289]. However, while widely used, the efficacy of anti-Gr1 depletion on both MDSC subtypes is controversial, as M-MDSCs express less Gr1 [296,297]. Although many of these therapeutics have been tested only in the context of cancer, they could also be applied to target sxMDSCs and could be tested for safety and efficacy using our murine model of surgical stress. It is important to note, however, that sxMDSCs may also play a role in homeostasis in response to postoperative inflammation, as they have recently been reported to be beneficial in the context of pregnancy, obesity, diabetes, and autoimmune disorders [298].

## 9. Acquitted: Surgery-Independent Mechanisms Anesthesia, Analgesia, and Blood Transfusions

Surgical stress results in profound and complex postoperative changes. These changes directly and indirectly, through cellular immune suppression, affect cancer cell growth, proliferation, and migration leading to increased metastasis and cancer recurrence. However, other perioperative interventions may also have an impact on postoperative metastasis. Anesthesia, analgesia, and blood transfusions are all known to modulate the inflammatory response and have recently been shown to impact cancer progression [110].

## 10. Double Agents? Anesthesia and Analgesia Having Varying Effects on Postoperative Metastasis

Propofol is the most commonly used intravenous anesthetic for induction and maintenance of anesthesia, while volatile agents, such as sevoflurane and isoflurane, are also commonly used for maintenance of anesthesia during surgery. Propofol has been shown to have anti-cancer properties as well as the potential to boost NK cell function. Zhou and colleagues investigated the effects of propofol on NK cell phenotype and function in patients with esophageal squamous cell carcinoma and found that propofol enhanced NK cell cytotoxicity through the expression of germline receptors [232]. Additional studies of co-culture models with the the colon cancer cell line SW620 [299] and the gastric cancer cell line BGC-823 [300] have also shown that propofol promotes NK cell cytotoxicity *in vitro.* Thus, propofol’s anti-cancer effects may be mediated by enhanced NK cell killing, although other hypothesized mechanisms include inhibition of oncogenes and the downregulation of HIF-1α and subsequent inhibition of angiogenesis [301].

As compared to propofol, the literature on the effects of volatile anesthetics on NK cell function is mixed. Lim et al., assessed the effects of propofol versus sevoflurane on NK cells isolated from patients who underwent breast cancer surgery. They found no difference in NK cell numbers or in cancer cell apoptosis and NK cell-derived cytokines when cultured *in vitro* with a breast cancer cell line [302]. However, Tazawa and colleagues assessed NK92-MI cytotoxicity, proliferation, conjugation, lytic granule polarization, and degranulation in a co-culture system with K562 cells and found that isoflurane and sevoflurane attenuated cytotoxicity, conjugation, and polarization of NK cells via LFA-1 inhibition [303]. Additionally, in a prospective randomized clinical study of patients undergoing breast cancer surgery, isolated NK cell cytotoxicity increased compared to baseline in patients who had received propofol-ketorolac compared to a decrease in cytotoxicity seen in patients that had received sevoflurane-fentanyl [304]. There even appears to be differences between volatile anesthetics in terms of their effects on NK cell function. Markovic et al., assessed interferon stimulation of NK cells in mice anesthetized with isoflurane versus halothane and found that while both anesthetics inhibited NK cell cytotoxicity, halothane induced more profound suppression [305]. In addition to their immunosuppressive effects, volatile agents have also been shown to stimulate proliferation and migration of cancer cells *in vitro* [110], in addition to inducing HIF-1α expression [306], therefore promoting cancer recurrence. Thus, there is much left to be uncovered about the effects of anesthetics on NK cell function and the role of anesthetics in cancer recurrence. As a result, numerous clinical trials are currently underway to address these unanswered questions [149,307].

Two commonly used classes of analgesics include opioids and NSAIDs. Opioids are potent analgesics that can also be used as anesthetic adjuncts perioperatively. In contrast to NSAIDs, the majority of preclinical studies suggest that opioids have pro-cancer effects [110,117] and retrospective reviews have associated opioid administration with increased cancer recurrence [110]. Opioids have been shown to suppress NK cell cytotoxicity via multiple mechanisms, including stimulation of the HPA axis, thereby inhibiting NK cell function through the release of immunosuppressive GCs, and the inhibition of NK cell migration via mu receptor activation [196]. Moreover, Shavit et al., used a rat lung tumor model to show that fentanyl (0.1–0.3 mg/kg) induced a dose-dependent increase in lung tumor retention and lung metastases [308]. Finally, a study by Desmond et al., reported reduced NK cell infiltration into breast cancer tissue in women who received more systemic perioperative opioids [309].

There is growing evidence to support the anti-cancer effects of COX-inhibiting NSAIDs, which act by reducing prostaglandin production. Preclinical studies have reported reduced cancer cell viability, proliferation, and migration through COX-dependent and -independent mechanisms while animal models suggest potential mechanisms may include reduced VEGF and the downregulation of oncogenes [310,311]. In a retrospective clinical study, combined NSAID (Flubiprofen Axetil)-dexamethasone resulted in enhanced survival of NSCLC patients [44] and is currently being investigated in a Phase 4 clinical trial (NCT03172988). However, these anti-cancer mechanisms are likely independent of innate immunity, as NSAIDs have been shown to suppress innate NK cell cytokine secretion (IFNγ, TNFα) in a COX-independent manner [170].

Thus, different anesthetics and analgesics have varying pro- and anti-cancer effects. Due to this variation and a lack of standardization for anesthetic/analgesic use in the perioperative period, the administration of anesthetics/analgesics perioperatively is unlikely to be the unifying mechanism responsible for metastatic recurrence across all cancer surgery patients.

## 11. Copycat Crime: Blood Transfusions Suppress Postoperative NK Cell Activity

The transfusion of RBCs and other blood products may be required during surgery. Blood transfusions have consistently been associated with immunosuppression and inflammation *in vitro* [312,313,314,315]. In the context of “pure” RBC transfusions, transfusion-related immunomodulation (TRIM) is thought to be mediated by monocytes/macrophages [313]. Specific to NK cells, in 1992, Jensen et al., assessed NK cell function preoperatively and postoperatively on POD3, 7, and 30 in elective CRC surgery patients. They found that, compared to patients that did not receive a transfusion or received filtered blood free from leukocytes, patients transfused with whole blood had significantly impaired NK cell cytotoxicity via ^51^Cr-release assay up to POD3 [314]. Similarily, Ghio and colleagues found that NK cells isolated from patients who received whole blood transfusion had reduced cytotoxic activity on day 3, which improved by day 7 after transfusion [315]. Clinically, blood transfusions have been associated with increased cancer recurrence, postoperative infection, and increased morbidity in a variety of cancer types, including gastric, esophageal, prostate, and bladder [316,317,318,319,320]. A 2006 Cochrane meta-analysis of CRC patients reported an overall odds ratio for recurrence of 1.42 in patients that received a blood transfusion (95% CI: 1.2–1.67) [321]. Interestingly, transfusion of autologous salvaged blood resulted in increased NK cell precursors and IFNγ production as compared to allogeneic transfusion [322]. However, the literature supports a consistent suppression of the cellular immune response in both transfused and non-transfused cancer surgery patients. This strongly suggests that although blood transfusions may contribute to postoperative immunosuppression and metastasis, it is not the primary mechanism.

## 12. The Verdict: Guilty

Surgery results in a paradoxical increase in metastasis and cancer recurrence and there exist numerous potential mechanisms that may contribute to this phenomenon. This process is likely multifactorial and may involve all of the potential mechanisms discussed here, however, evidence supports a primary role for postoperative immune suppression, specifically a suppression of NK cells. The inability of surgically stressed NK cells to carry out effector functions is devastating for the anti-tumor immune response.

Although currently there are no FDA-approved perioperative therapeutics, numerous pre-clinical and clinical investigations are aimed at identifying and testing such therapeutics. Novel therapies may target the many hypothesized mechanisms of postoperative metastasis, which indirectly result in immune suppression, or they may target suppressive cytokines and immune cell populations, or they may involve perioperative boosting of NK cell function. Specifically, studies targeting soluble factors and suppressive cell populations during the anti-inflammatory phase of the postoperative period have shown promising results. It will be critical to apply the previously discussed immunotherapeutics in an NK cell-specific manner, as the disturbance of perioperative processes may also impact wound healing and patient recovery. The perioperative period of immunosuppression presents a window of opportunity for such therapeutics to prevent metastases and cancer recurrence for thousands of cancer patients who undergo tumor resection each year.

## Figures and Tables

**Table 1:** Potential therapeutics to target postoperative Natural Killer cell suppression.

Mechanism	Potential Target	Potential Therapies	Potential Adverse Surgical Effects
Tissue Hypoxia	NK cells	Preoperative IL-2 adminsitration Autologous genetic engineering of NK cells to produce IL-2 endogenously	Increased risk of systemic inflammation and hypercytokinemia
Neurendocrine activation	β-adergenic receptor	Propranolol	Cardiopulmonary effects
	Glucocorticoid receptor	Mifepristone	Severe hypokalemia, hypertension, and adrenal insufficiency
Hypercoagulable state	Coagulation	Low-molecular weight heparin (LMWH)	Thrombocytopenia, leading to increased risk of internal bleeding
Pro-inflammatory phase/prostaglandins	Prostaglandins	NSAIDs	Potentially suppress NK cell cytokine secretion
	COX-2	RQ-15986	No clinical data currently available
Anti-inflammatory phase			
*Soluble factors*			
IL-6	IL-6 IL-6R (α, gp130)	Ligand trap (siltuximab) Receptor blockade (tocilizumab, raloxifene)	Little improvement in clinical outcomes
	JAK	Inhibition of signal transduction (ruxolitinib)	
IL-10	IL-10	Ligand trap (BT063)	Pleitropic effects render it ineffective in targeting postoperative metastasis
TGFβ	TGFβ TGFβRII STAT3	Ligand trap (fresolimumab) Receptor blockade (LY3022859) Inhibiting signal transduction (BBI608 [116], Celecoxib)	Impaired wound healing Observed cardiac valvular toxicity
*Suppressive cell populations*			
T_regs_	T_regs_ (depletion)	Cyclophosphamide	Anemia, impaired wound healing
		Lenalidomide/pomalidomide	Risk of thrombocytopenia and deep vein thrombosis
	Immune checkpoints	Combition anti-PD-1 and anti-CTLA-4	Systemic inflammation/autoimmunity
MDSCs	ARG-1, iNOS, COX-2 ARG-1 STAT3	nor-NOHA, sildenafil, tadalafil, anti-PD-1 with entinostat siRNA or decoy oligonucleotides	Systemic inflammation/autoimmunity Increased risk of heart failure
	MDSC TME migration	Small molecule inhibitors or chemotherapeutic drugs (reparixin, MK7123)	
	MDSCs (depletion)	Dimethyl amiloride, omeprazole	
		All-trans retinoid acid, docetaxel Sunitinib Gemcitabine and 5-fluorouracil	
